# Causes of Death in Acute Pneumonia

**Published:** 1881-09

**Authors:** 


### CAUSES OF DEATH IN ACUTE PNEUMONIA.

It may be well to state that I recognize three varieties of acute pneumonia:

First.—A variety in which the primary and principal local pathological changes begin in, and are mainly confined to, the vesicular structure of the lungs, called croupous or lobar pneumonia.

Second.—A variety in which the primary local pathological changes begin in the pleura, the vesicular structure of the lungs being only secondarily involved, called pleuro-pneumonia.

Third.—A variety in which the primary local pathological changes begin in the bronchi, the vesicular structure of the lungs being slowly and gradually involved, called catarrhal or broncho-pneumonia.

After the pneumonic process is fully established, it is often difficult, and sometimes (from a clinical standpoint) impossible to distinguish these different varieties, unless careful and intelligent observations have been made of the mode of their accession. I shall this evening restrict the term pneumonia to those varieties in which the primary local pathological lesion consists in a fibrinous and cell exudation poured out upon the free surface of the lining membrane of the bronchioles, the alveoli, and the pleura.

In seeking for the causes of death in pneumonia, observers have taken the results of their *post-mortems* as a standard of their observations—one finds œdma of the lungs at the majority of his autopsies; another finds a clot in the heart in most of his fatal cases; hence the conclusion is reached that pulmonary œdma and heart-clots are the causes of death in pneumonia. But in every disease there is a great difference between the *cause* and *mode* of death. If, as a result of the failure of heart-power during the last hours of life, pulmonary congestion and œdma are devolped and clots are found in the heart cavities, it cannot be assumed that these conditions are the causes of the death. Jurgensen states that in fatal cases of pneumonia, œdma of the lungs is probably *always* present, and heart-clots are frequently met with.

The majority of writers make mention of certain diseases which may be regarded as complications of pneumonia, while but few give a condensed statement of their statistical bearing upon the death-rate By a careful study of these complications it is apparent that they all exert a direct influence on the heart, diminishing its power and crippling its action by obstructing the blood current from the right ventricle toward the lungs. It is unnecessary to discuss these complications in detail; it is sufficient to state that weakening of the contractile power of the cardiac muscle is an essential feature of endocarditis, pericarditis, Bright's disease and alcoholism. In all the acute infectious diseases, such complications are regarded as dangerous, because they increase the sources of heart-failure where such failure is especially to be found.

At this point the question meets us, Is pneumonia a specific constitutional disease or a local inflammation? Jurgensen gives very positive answers to this question: he states without qualification that pneumonia is a constitutional disease, and never dependent upon a local cause; that the pulmonary inflammation and the morbid phenomena which attend it are not due to the local affection; that it belongs to the class of acute infectious diseases; that it cannot be produced by any of the usual causes of inflammation, however strong or weak their action; and that, as in typhoid fever, there must be a specific exciting cause.

I am not prepared to accept these unqualified statements of its specific character. It seems to me to occupy a middle ground between infectious diseases and local inflammations, having some elements which are common to both. It resembles infectious diseases in its initiatory chill, in its orderly pyrexia, in its critical days, and in its definite duration. No local disease runs a general typical course. The etiological conditions under which it is developed closely ally it to infectious diseases. The influence which miasmatic and septic emenations, as well as certain atmospheric conditions, exert in the development of pneumonia, are well recognized. The " sewer-gas pneumonia," which has been of so frequent occurrence in this city during the past few years, is an example of this. In its etiology, in many instances, it seems to be very similar to cerebro-spinal meningitis and diphtheria. The mode of its invasion in adults in its severe forms resembles very closely the invasions of some of the severer forms of infectious fever. The convulsions occurring in the very young, and coma and collapse in the very old, before the hepatization is completely established, allies it those infectious diseases in which a specific and morbific agent acts primarily and principally on the nervous system. Its anatomical changes are entirely distinct from those of any other inflammation and this allies it to the inflammations which occur in the course of cerebro-spinal meningitis and diphtheria.

Pneumonia differs, however, from infectious diseases in that it has no prodromata, and no known period of incubation; it is not contagious, and it is is not epidemic. It has no typical range of temperature, no constant and characteristic surface phenomena. It is not a fever which, apart from any fixed seat, pervades the system generally. At one time it will present almost exclusively the character of a local disease, and at another chiefly those of a specific fever. Dr. Wilson Fox says that the disease which pneumonia most resembles in its clinical phenomena is quinsy and erysipelas. Dr. Dickinson finds the evidence of a post-mortem relationship between pneumonia and acute tubal nephritis. Dr. Sturgis sees a similarity between pneumonia and rheumatic fever. Other observers speak of the close clinical and pathological resemblance of pneumonia to acute infectious diseases. If we carefully examine the points of resemblance between it and these diseases we shall find in the majority of instances that the resemblance lies in the nervous phenomena which attend its development. As has been shown, the complications which render pneumonia dangerous either interfere directly with the muscular power of the heart, or diminish its nerve-supply. In order in some degree to determine the influence which a diminished or vitiated nerve-supply may have upon the contractile power of the heart, I shall briefly refer to a few well established physiological facts.

Few clinical observers of large experience question that death in pneumonia is due in the majority of instances to insufficiency of the heart. I have endeavored to show the different views that are entertained as to the causes of this insufficiency. But my own experience leads me to the opinion that, in a large number of cases, it can only be accounted for satisfactorily on the basis of a defective nerve-supply to the heart. This nerve-failure may be due to the presence in pneumonia of a morbific agent (as in certain infectious diseases), which so acts upon the nerve-centers which supply the heart that its contractile power is diminished, and its rythm disturbed. In a certain proportion of cases there undoubtedly exists, prior to the occurrence of pneumonia, a weakened or perverted condition of the nervous system (such as is to be met with in chronic alcoholism, chronic uræmia, and in the old and feeble), in which the cardiac nerve supply is easily disturbed, and consequently the signs of heart-failure appear early and are excessive.

Taking the ground, then, that the failure of nerve-power may show itself at the very onset of a pneumonia, and that the heart may commence to stagger at the very commencement of the attack, it is evident that the effect of the local pneumonic changes is to increase the elements of heart-failure; for the pneumonic consolidation causes an increased resistance to the pulmonary circulation, and consequently calls for increased power on the part of the right ventricle. Then the surfaces over which the blood and the air come in contact in the lungs are diminished by the pneumonic exudation, and yet another cause of obstruction to the pulmonary circulation is developed. Again, fever induces increased labor on the part of the heart. Thus, from all sides, the heart is the organ upon which the burden of the obstructed pulmonary circulation is thrown. If, in addition to these obstructions to the pulmonary circulation from the pneumonic infiltration, there shall occur a crippling of the heart from endocardial or pericardial inflammation, or if extensive pleuritic inflammation shall restrict the respiratory movements, the chances of early and fatal heart-failure are greatly increased.

The importance of detecting the first signs of commencing heart-failure is apparent, and should lead one to a careful examination of each pulsation. At first, it will be noticed that the individual pulsations produce a variable filling of the arteries with blood, the arterial beats, as felt by the finger, vary in force; then occur waves; and then true intermissions. However significant a rapid pulse may be, I regard intermission, or even mere irregularity in the filling of the arteries, as far more important indications of a dangerous heart-failure, especially if it is attended by an entire loss or marked feebleness of the muscular element of the first sound of the heart. If, then, as I have endeavored to show, deficient or vitiated nerve-supply is the great cause of heart insufficiency in pneumonia, and if this insufficiency begins much earlier than is usually recognized by our ordinary methods of examination—for in many instances I have been able to detect it within twenty-four hours from the commencement of the pneumonic attack, occasionally, even during the initiatory chill, due, as it seems to me, to the shock which marks the advent of the disease—the question arises, What measures shall we employ to overcome or mitigate the impression made upon the nervous system by the morbific agent which is operating to produce the pneumonia?

The experience of the past eighteen months leads me to state with some positiveness that in opium we have such an agent. My rule, for the past year, has been to bring my patient under the full influence of the drug at the onset of a pneumonia, and to hold him in a condition of comparative comfort until the pneumonic infiltration is completed (usually for the first four days of the disease). After this period, the greatest care must be exercised in its use, for now a new danger threatens, namely, paralysis of the bronchi, and consequent accumulation of secretion in the bronchial tubes, which will greatly increase the difficulties of respiration; but during the developing period of the disease, when the pneumonic blow is first struck, morphia hypodermically seems to lessen the nervous shock and to diminish or prevent the effect of the pneumonic poison on the nervous system, until the first violence of the poison has been spent in completing the pulmonary infiltration. The use of opium in this way does not interfere with the usual anti-pyretic treatment of the disease, nor does a demand for alcoholic stimulants contra-indicate its use. The results which have followed this plan of treatment in the limited number of cases in which I have been able to fairly test it (in patients that have been directly under my personal management) have convinced me that it greatly diminishes the chances of heart-failure, and cases which from their age and attending circumstances seemed hopeless, have recovered.

The great relief and comfort which the use of opium in this way gives to the pneumonic sufferer during the first four days of his struggles, are sufficient to commend it especially in those cases where an extensive pleuritic inflammation accompanies the pneumonic development.—*A. L. Loomis, in Medical Record.*

